# Nasal inflammation and its response to local glucocorticoid regular treatment in patients with persistent non-allergic rhinitis: a pilot study

**DOI:** 10.1186/s12950-016-0134-3

**Published:** 2016-08-04

**Authors:** Donatella Poletti, Valeria Iannini, Paolo Casolari, Marco Contoli, Alberto Papi, Paul Kirkham, Trevor T. Hansel, Kian Fan Chung, Peter J. Barnes, Antonio Pastore, Stefano Pelucchi, Ian M. Adcock, Gaetano Caramori

**Affiliations:** 1 ORL, Azienda USL Ferrara, Italy, Sezione di Scienze Otorinolaringoiatriche e Fisica Medica, Departimento di Scienze Biomediche e Chirurgico Specialistiche, University of Ferrara, Ferrara, Italy; 2 Department of Biomedical Sciences, Faculty of Science and Engineering, University of Wolverhampton, Wolverhampton, UK; 3 Airway Disease Section, National Heart and Lung Institute, Imperial College London, London, UK; 4 Centre for Respiratory Infection, National Heart and Lung Institute at Imperial College, St. Mary’s Hospital, Mint Wing, Entrance C, Paddington, London, W2 1NY UK; 5 Centro Interdipartimentale per lo Studio delle Malattie Infiammatorie delle Vie Aeree e Patologie Fumo-correlate (CEMICEF), Dipartimento di Scienze Mediche, Sezione di Medicina Interna e Cardiorespiratoria, Università di Ferrara, Via Ludovico Ariosto 35, 44121 Ferrara, Italy; 6 Sezione di Scienze Otorinolaringoiatriche e Fisica Medica, Dipartimento di Scienze Biomediche e Chirurgico Specialistiche, University of Ferrara, Ferrara, Italy

**Keywords:** Non-allergic rhinitis, Nasal scraping, Nasal inflammation, Nasal epithelial cells, Goblet cells, MUC5AC, p65, Eosinophils

## Abstract

**Background:**

The pathogenesis of non-allergic rhinitis (NAR) is still largely unknown. Furthermore, it is unclear whether there is a correlation between the effect of nasal glucocorticoids on nasal inflammation and on nasal symptoms and quality of life.

**Methods:**

In this pilot study we recruited 12 healthy subjects and 24 patients with recently diagnosed persistent NAR [12 untreated and 12 under regular treatment with nasal fluticasone furoate (two sprays of 27.5 μg each in each nostril once daily, total daily dose = 110 μg) for at least 20 days]. Each subject filled a mini rhinoconjunctivitis quality of life questionnaire (mini RQLQ). Nasal scrapings were obtained from each subject and used to prepare slides for Diff-Quik and immunocytochemical staining for inflammatory and epithelial cells count, MUC5AC expression and the general pro-inflammatory transcription factor nuclear factor kB (NF-kB) activation.

**Results:**

The nasal score of the mini RQLQ, the number of nasal inflammatory cells (neutrophils, eosinophils) and the number of goblet cells are significantly higher in untreated patients with persistent NAR compared with control subjects and treated NAR patients. The percentage of MUC5AC+ nasal epithelial cells is significantly increased in untreated patients with persistent NAR compared with the control subjects (41.8 ± 6.4 vs 22.3 ± 4.8, respectively; *p* = 0.0403) without significant differences between control subjects and patients with persistent NAR on regular fluticasone furoate treatment (33.9 ± 5.0 %; *p* = 0.0604) nor between the 2 groups of persistent NAR subjects (*p* = 0.3260). The number of cytosolic and/or nuclear p65+ nasal epithelial and inflammatory cells was not significantly different between the three groups.

**Conclusions:**

Patients with persistent untreated NAR, compared with normal control subjects and patients with persistent NAR under regular treatment with nasal fluticasone furoate by at least 20 days, have more nasal symptoms, worst quality of life and an increased number of nasal inflammatory cells (neutrophils, eosinophils), goblet cells and MUC5AC+ nasal epithelial cells. This nasal inflammation seems unrelated to NF-kB activation.

## Background

Non-allergic rhinitis (NAR) is a term that can be applied to any disease of the nose presenting with obstructive and secretory symptoms, with or without hyperirritability, that does not have a known allergic [immunoglobulin (Ig)E-mediated)] etiology. In fact, the disease is “non-allergic” when allergy has not been proven by proper allergy examination (history, skin prick testing, measurement of serum specific IgE antibodies) [1]. This definition can be narrowed by allowing only chronic conditions to be included and, therefore, by excluding acute viral and acute bacterial infections [1]. The pathogenesis of persistent (chronic) NAR is still largely unknown [2].

Nasal cytology, obtained using nasal scraping, is a promising, scarcely invasive, tool to investigate nasal inflammation, but most of the studies have been performed so far in patients with allergic rhinitis only [3]. The aim of this study was to investigate, using nasal cytology, inflammatory cell counts, the activation of the general pro-inflammatory transcription factor nuclear factor kB (NF-kB) and the expression of MUC5AC (the main secretory mucin expressed on the surface of the nasal epithelium), in patients with untreated persistent NAR and a control group of normal subjects and to investigate the response to the regular treatment with endonasal glucocorticoids, the gold standard treatment, in a separate cohort of patients with persistent NAR under regular treatment with nasal glucocorticoids by at least 20 days. We also have correlated these results with the quality of life of the subjects using a validated questionnaire.

## Methods

The study was approved by the local ethics committee of the University Hospital of Ferrara, Italy (www.ospfe.it), and informed consent was obtained from each participant in accordance with the principles outlined in the Declaration of Helsinki.

We recruited 3 groups of subjects, aged between 18 and 65, all lifelong non-smokers, nonatopic [negative skin prick tests and/or RAST for the most common aeroallergens in Italy (house-dust mites (Dermatophagoides pteronyssinus and farinae), cat and dog dander, plant pollens (grass mix, Parietaria, Olea europaea, Cupressus sempervirens, Betula pendula, Corylus avellana, Artemisia vulgaris) and molds (Alternaria tenuis, Aspergillus fumigatus and Cladosporium herbarum)] and non-asthmatic. Their demographic characteristics are summarised in Table 1. There is a significant difference in the mean age between the healthy control group and the patients with NAR + nasal fluticasone furoate (FF) (32.5 ± 2.7 vs 48.1 ± 4.0, respectively; 95 % confidence interval 26.6─38.4 vs 39.2─56.9, respectively; Mann–Whitney U test *p* = 0.0091).

**Table 1:** Study subjects demographic characteristics

	*n*	Age	Sex	Mini-RQLQ score
Healthy control group	12	32.5 ± 2.7	4 M/8 F	8.1 ± 4.4
Nonallergic persistent rhinitis	12	39.5 ± 3.7	4 M/8 F	32.9 ± 5.6
Nonallergic persistent rhinitis + nasal FF	12	48.1 ± 4.0	5 M/7 F	15.7 ± 4.7

The healthy subjects in the control group (*n* = 12, mean age 32.5 ± 2.7; 4 M/8 F) were all non-rhinitics and free from any other nasal disease. A group (*n* = 12) of patients were recruited with persistent untreated non-allergic rhinitis (mean age 39.5 ± 3.7; 4 M/8 F). None of these patients or control subjects had been treated in the least 60 days with systemic and/or nasal glucocorticoids, anti-histamine 1 receptor drugs, anti-leukotrienes, methylxanthines, or any kind of immunosuppressing drugs, mucolytics/antioxidants drugs by systemic or local routes.

A separate second cohort (*n* = 12) of patients with chronic non-allergic rhinitis (mean age 48.1 ± 4.0; 5 M/7 F) were treated regularly for at least 20 days with nasal fluticasone furoate (two sprays of 27.5 μg each, in each nostril once daily, total daily dose = 110 μg) but not anti-histamine 1 receptor drugs and/or anti-leukotrienes, methylxanthines, or any kind of other immunosuppressing drugs, mucolytics/antioxidants drugs by systemic or local routes. All NAR patients in both groups were initially diagnosed within 12 months of their recruitment to the study.

Both groups of patients with persistent non-allergic rhinitis have been previously investigated with nasal endoscopy and maxillofacial computed tomography to exclude the presence of rhinosinusal polyposis and all these patients have never been previously treated with nasal or sinus surgery.

Each subject recruited to the study completed the mini rhinoconjunctivitis quality of life questionnaire (mini RQLQ) [4] (the permission to use the Italian validated version of this questionnaire was kindly given to GC by Prof. Elizabeth Juniper; https://www.qoltech.co.uk/mini_rqlq.html) (Table 2 in supplementary material).

**Table 2:** Italian validated version of the Mini Rhinoconjunctivitis Quality of Life Questionnaire (MiniRQLQ)

	Nessun fastidio	Quasi nessun fastidio	Un leggero fastidio	Poco fastidio	Abbastanza fastidio	Molto fastidio	Moltissimo fastidio
*Attività*							
l. Attività regolari a casa e al lavoro	0	1	2	3	4	5	6
(il lavoro o le faccende che deve svolgere regolarmente in casa)							
2. Attività sociali	0	1	2	3	4	5	6
(*es*., attività con la famiglia e gli amici, giocare con i bambini e gli animali domestici, rapporti sessuali, passatempi)							
3. Attività all'aperto	0	1	2	3	4	5	6
(*es*., giardinaggio, tagliare il prato, stare seduti all'aperto, praticare sport, fare una passeggiata)							
*Sonno*							
4. Difficoltà a prendere sonno	0	1	2	3	4	5	6
5. Svegliarsi durante la notte	0	1	2	3	4	5	6
6. Mancanza di una buona dormita	0	1	2	3	4	5	6
*Problemi generali*							
7. Affaticamento (mancanza di energia)	0	1	2	3	4	5	6
8. Sete	0	1	2	3	4	5	6
9. Produttività ridotta	0	1	2	3	4	5	6
10. Sentirsi assonnato/a	0	1	2	3	4	5	6
11. Scarsa concentrazione	0	1	2	3	4	5	6
12. Mal di testa	0	1	2	3	4	5	6
13. Sfinimento, spossatezza	0	1	2	3	4	5	6
*Problemi pratici*							
14. La scomodità di dover portare con sé	0	1	2	3	4	5	6
fazzoletti o fazzolettini di carta							
15. Il bisogno di strofinarsi il naso o gli occhi	0	1	2	3	4	5	6
16. Il bisogno di soffiarsi ripetutamente il naso	0	1	2	3	4	5	6
*Disturbi nasali*							
17. Naso chiuso	0	1	2	3	4	5	6
18. Naso che cola	0	1	2	3	4	5	6
19. Starnuti	0	1	2	3	4	5	6
20. Catarro in gola	0	1	2	3	4	5	6
*Disturbi agli occhi*							
21. Prurito agli occhi	0	1	2	3	4	5	6
22. Occhi che lacrimano	0	1	2	3	4	5	6
23. Occhi irritati	0	1	2	3	4	5	6
24. Occhi gonfi	0	1	2	3	4	5	6
*Aspetti emotivi*							
25. Si è sentito/a contrariato/a e deluso/a	0	1	2	3	4	5	6
26. Impazienza e inquietudine	0	1	2	3	4	5	6
27. Irritabilità	0	1	2	3	4	5	6
28. Imbarazzo a causa dei disturbi	0	1	2	3	4	5	6

Juniper et al. [4]. The permission to use this Italian validated version of the questionnaire has been kindly allowed to Prof. Gaetano Caramori by Prof. Elizabeth Juniper; https://www.qoltech.co.uk/mini_rqlq.html

### Nasal scrapings and nasal cytology

Each subject recruited to the study underwent a single nasal scraping. The nasal scraping was performed under direct visual inspection using a plastic curette in the middle third of the inferior turbinate, an area previously reported to have an optimal ratio between ciliated and mucous-secreting cells, usually in favour of ciliated cells [5]. There is no single published standardized protocol for processing these samples and for this reason we have followed the guidelines for sputum processing.

Nasal scrapes were immediately put onto ice and maintained at 4° ˚C throughout their processing. Each nasal scraping was processed by cytocentrifugation, to obtain at least 4 cytospins from each subject. Cytospin slides were prepared and dried for 30 min. An average of at least six slides were prepared from each patient. Two of these cytospins were stained using Diff-Quik method exactly according to the Manufacturer’s instructions (Dade Behring, Milan Italy) for total and differential cell counts. The remaining slides were wrapped in aluminum foil and stored at −20 °C before immunocytochemical staining for MUC5AC and p65.

### Immunocytochemistry for MUC5AC and p65 in nasal cytospins

We used a protocol previously described [6]. Nasal cytospins were fixed with 4 % paraformaldehyde (PFA, 10 min, 22 °C), washed (PBS 1X, twice for 5 min, 22 °C), and cells permeabilized (acetone/methanol, 10 min, −20 °C). The cell endogenous peroxidase activity was quenched (3 % hydrogen peroxide, 30 min, 22 °C). Non-specific binding was blocked (5 % normal horse serum or 5 % normal goat serum, 20 min, 22 °C) before incubation with the specific mouse anti-MUC5AC clone 45 M1 (sc-21701, Santa Cruz Biotechnology, USA; 1:1100 dilution, 1 h, 22 °C) or rabbit anti-p65 (sc-372, Santa Cruz Biotechnology, USA; 1:100 dilution, 1 h, 22 °C). The selectivity of both these primary antibodies has been previously described [6, 7]. We did not performed the immunocytochemical staining for MUC5B because in a separate pilot study we did not observe any MUC5B+ staining in nasal epithelial cells from either normal subjects or patients with persistent untreated or treated NAR using appropriate positive controls and a validated primary antibody [8].

Both MUC5AC and p65 were detected using peroxidase (Vectastain Elite ABC-Peroxidase Kit Standard, Vector Laboratories, Burlingame, CA, USA) and DAB substrate according to the Manufacturer's instructions. Negative control slides (nonspecific immunoglobulin of the same isotype of the primary antibody) were included in each staining run for each patient for every group of subjects (control subjects, untreated and treated patients with persistent NAR).

### Cell count

Cell counts were performed on the Diff-Quik-stained slides using an optical microscope at a x1000 total magnification, by two independent blinded observers (PC and VI). The mean intra-observer coefficients of variance with counting were less than 10 %. To have a more complete data set we counted all cells within specific grids on each slide (total number of cells), distinguishing and counting inflammatory and nasal epithelial cells, the different subsets of inflammatory cells and the number of goblet cells among the nasal epithelial cells.

Goblet cells were defined as glandular, modified simple columnar epithelial cells [9].

The total cell count was standardized for the sample size (ml) as previously described [6].

All MUC5AC+ and p65+ cells staining brown, indicating MUC5AC or p65 immunoreactivity, were counted on each slide. p65+ cells were also quantified for cytoplasmic and/or nuclear (an index of activation) staining.

### Statistical analysis

Group data were expressed as mean and standard error (SEM). The Kruskal-Wallis test for nonparametric data followed by Mann–Whitney U test was used to determine differences between the three groups of subjects and the post-test Dunn’s comparison. A probability value of <0.05 was considered significant.

## Results

### Clinical parameters

**Fig. 1:**
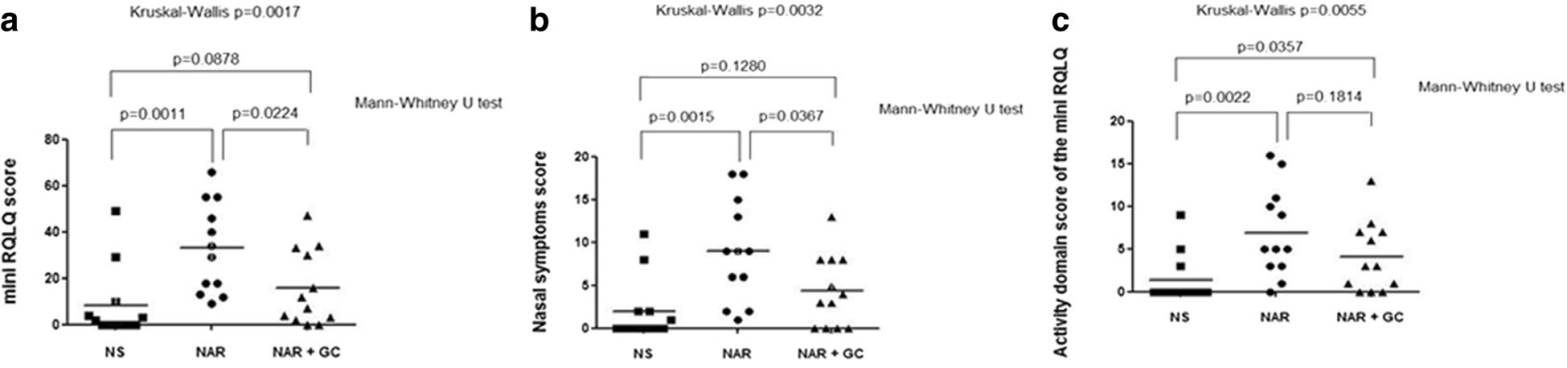
**a**) The mini RQLQ total score is significantly increased in the persistent untreated nonallergic rhinitis (NAR) patients compared both to control normal subjects (NS) and the persistent NAR patients under regular treatment with nasal glucocorticoids (NAR + GC), without any significant difference between control subjects and persistent NAR patients under regular treatment with nasal glucocorticoids. **b**) The nasal symptoms score of the mini RQLQ is significantly increased in the persistent untreated NAR patients compared both to NS and NAR + GC, without any significant difference between control subjects and persistent NAR patients under regular treatment with nasal glucocorticoids. **c**) The interference of rhinitis pathology with the daily activities score of the mini RQLQ is significantly increased both in the persistent untreated NAR patients and NAR + GC compared with NS, without any significant difference between both groups of persistent NAR patients

The total mini RQLQ score was significantly increased in the patients with untreated persistent NAR compared both to the control normal subjects and the patients with persistent NAR under regular treatment with FF (32.9 ± 5.6 vs 8.1 ± 4.4 vs 15.7 ± 4.7, respectively; 95 % confidence interval 20.6─45.3 vs −1.7─17.8 vs 5.4─26.0, respectively; Mann–Whitney U test p = 0.0011 and *p* = 0.0224, respectively; one-way ANOVA Dunn’s multiple comparison test NAR vs NAR + FF *p* > 0.05), without significant differences between control subjects and patients with persistent NAR under regular treatment with nasal FF (Mann–Whitney U test *p* = 0.0878) (Kruskal-Wallis test p = 0.0017) (Fig. 1a).

The nasal symptoms (sneezing, stuffy blocked nose, runny nose) score was significantly increased in the patients with untreated persistent NAR compared both to the control normal subjects and the patients with persistent NAR under regular treatment with nasal FF (9.0 ± 1.7 vs 2.0 ± 1.1 vs 4.3 ± 1.2, respectively; 95 % confidence interval 5.2─12.8 vs −0.3─4.3 vs 1.7─7.0, respectively; Mann–Whitney U test *p* = 0.0015 and *p* = 0.0367, respectively; one-way ANOVA Dunn’s multiple comparison test NAR vs NAR + FF *p* > 0.05), without significant differences between control subjects and patients with persistent NAR under regular treatment with nasal glucocorticoids (Mann–Whitney U test *p* = 0.1280) (Kruskal-Wallis test *p* = 0.0032) (Fig. 1b). Furthermore, all patients with persistent NAR (both untreated and treated) showed a statistically significant worse daily activity score compared to the control group of normal subjects (6.9 ± 1.5 vs 4.1 ± 1.2 vs 1.4 ± 0.8, respectively; 95 % confidence interval 3.6─10.3 vs 1.5─6.7 vs −0.4─3.2, respectively; Mann–Whitney U test *p* = 0.0022 and *p* = 0.0357, respectively; one-way ANOVA Dunn’s multiple comparison test control group vs NAR + FF *p* > 0.05) (Fig. 1c) although this score, considered as “the interference of rhinitis pathology with the daily activities”, was not significantly affected by topical FF therapy and was not different between the 2 groups of persistent NAR subjects (Mann–Whitney U test *p* = 0.1814) (Kruskal-Wallis test *p* = 0.0055).

### Total and differential nasal inflammatory cells count

**Fig. 2:**
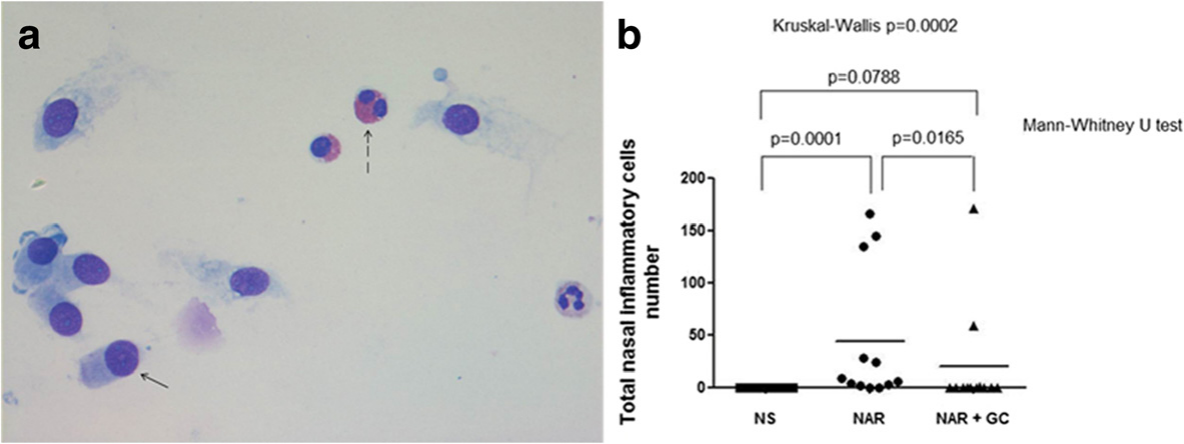
**a**) Diff-Quik staining of a nasal cytospin showing nasal ciliated epithelial cells (*arrow*) and nasal inflammatory cells (eosinophils and neutrophils) stained with Diff-Quik. The nuclei and cytosol of the epithelial cells and neutrophils are stained in *purple*/*blue*, cytosol of eosinophil granules is stained in *red* (*broken arrow*). Original magnification: x400. **b**) The total number of nasal inflammatory cells is significantly increased in the persistent untreated nonallergic rhinitis (NAR) patients compared both to control normal subjects (NS) and the persistent NAR patients under regular treatment with nasal glucocorticoids (NAR + GC), without any significant difference between control subjects and persistent NAR patients under regular treatment with nasal glucocorticoids

The total number of nasal inflammatory cells count (Fig. 2a) was significantly increased in patients with untreated persistent NAR compared both to the control normal subjects and patients with persistent NAR treated with nasal FF (43.5 ± 18.6 vs 0.0 ± 0.0 vs 19.3 ± 14.6, respectively; 95 % confidence interval 2.6─84.4 vs 0.0─0.0 vs −13.0─51.5, respectively; Mann–Whitney U test *p* = 0.0001 and *p* = 0.0165, respectively; two-way ANOVA Bonferroni multiple comparison column factor p = 0.1040). There were no significant differences between control subjects and NAR patients on nasal FF (Mann–Whitney U test *p* = 0.0788) (Kruskal-Wallis test *p* = 0.0002) (Fig. 2b).

**Fig. 3:**
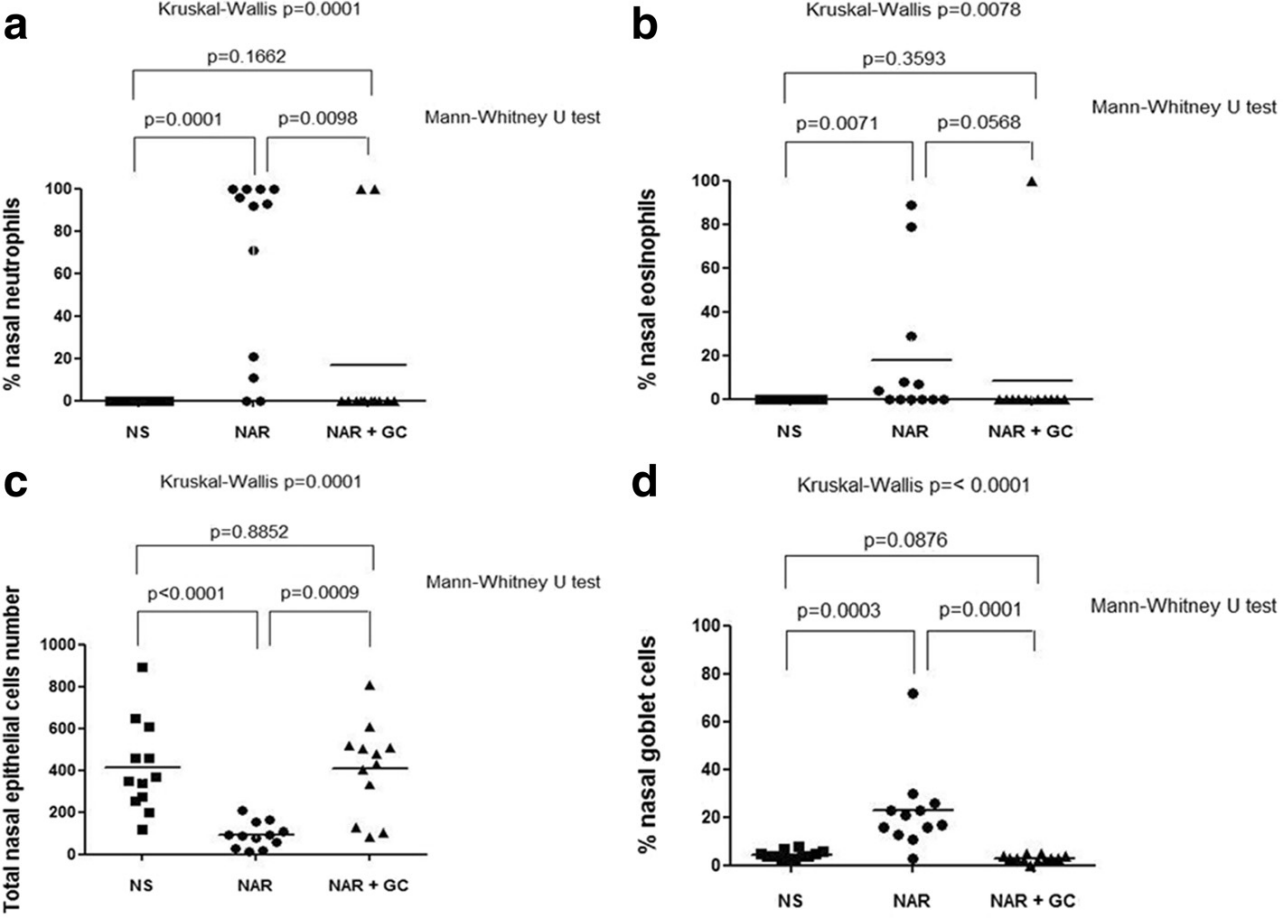
**a**) The percentage of neutrophils on total number of nasal inflammatory cells is significantly increased in the persistent untreated nonallergic rhinitis (NAR) patients compared both to control normal subjects (NS) and the persistent NAR patients under regular treatment with nasal glucocorticoids (NAR + GC), without any significant difference between control subjects and persistent NAR patients under regular treatment with nasal glucocorticoids. **b**) The percentage of eosinophils on total number of nasal inflammatory cells is significantly increased in the persistent untreated NAR patients compared both to NS and NAR + GC, without any significant difference between control subjects and persistent NAR patients under regular treatment with nasal glucocorticoids. **c**) The total number of nasal epithelial cells is significantly increased in the persistent untreated NAR patients compared both to NS and NAR + GC, without any significant difference between control subjects and persistent NAR patients under regular treatment with nasal glucocorticoids. **d**) The percentage of goblet cells on the total number of nasal epithelial cells is significantly increased in the persistent untreated NAR patients compared both to NS and NAR + GC, without any significant difference between control subjects and persistent NAR patients under regular treatment with nasal glucocorticoids

The percentage of nasal neutrophils was significantly increased in patients with untreated persistent NAR compared both to the control normal subjects and NAR patients on nasal FF (65.3 ± 12.5 vs 0.0 ± 0.0 vs 16.7 ± 11.2 %, respectively; 95 % confidence interval 37.8─92.9 vs −0.0─0.0 vs −8.1─41.4, respectively; Mann–Whitney U test *p* = 0.0001 and *p* = 0.0098, respectively) (Kruskal-Wallis test *p* = 0.0001) (Fig. 3a).

The percentage of nasal eosinophils was also significantly increased in the patients with untreated persistent NAR compared to the control normal subjects (18.0 ± 9.2 vs 0.0 ± 0.0 %, respectively; 95 % confidence interval −2.3─38.3 vs −0.0─0.0, respectively; Mann–Whitney U test *p* = 0.0071; two-way ANOVA Bonferroni multiple comparison column factor *p* = 0.2569) without significant differences between control subjects and patients with persistent NAR under regular treatment with nasal FF (8.3 ± 8.3 %; 95 % confidence interval −10.0─26.7; Mann–Whitney U test *p* = 0.3593) nor between the 2 groups of persistent NAR subjects (Mann–Whitney U test *p* = 0.0568) (Kruskal-Wallis test *p* = 0.0078) (Fig. 3b).

### Total nasal epithelial cells and nasal goblet cells count

The total number of nasal epithelial cells count was significantly different between patients with persistent untreated NAR compared both to the control normal subjects and NAR patients treated with nasal FF (92.3 ± 17.2 vs 413.8 ± 62.8 vs 407.8 ± 62.6, respectively; 95 % confidence interval 54.4─130.2 vs 275.7─551.9 vs 270.0─545.7, respectively; Mann–Whitney U test *p* < 0.0001 and *p* = 0.0009, respectively) with no significant differences between control subjects and nasal FF-treated NAR patients (Mann–Whitney U test *p* = 0.8852) (Kruskal-Wallis test *p* = 0.0001) (Fig. 3c).

The percentage of nasal goblet cells was significantly enhanced in untreated NAR patients compared to either control normal subjects and NAR patients treated with nasal FF(22.6 ± 5.0 vs 4.4 ± 0.6 vs 3.0 ± 0.4 %, respectively; 95 % confidence interval 11.7─33.5 vs 3.2─5.6 vs 2.1─3.9, respectively; Mann–Whitney U test *p* = 0.0003 and *p* = 0.0001, respectively). No significant differences between control subjects and nasal FF-treated NAR patients was observed (Mann–Whitney U test *p* = 0.0876) (Kruskal-Wallis test *p* < 0.0001) (Fig. 3d).

### Nasal epithelial cells MUC5AC+ count

**Fig. 4:**
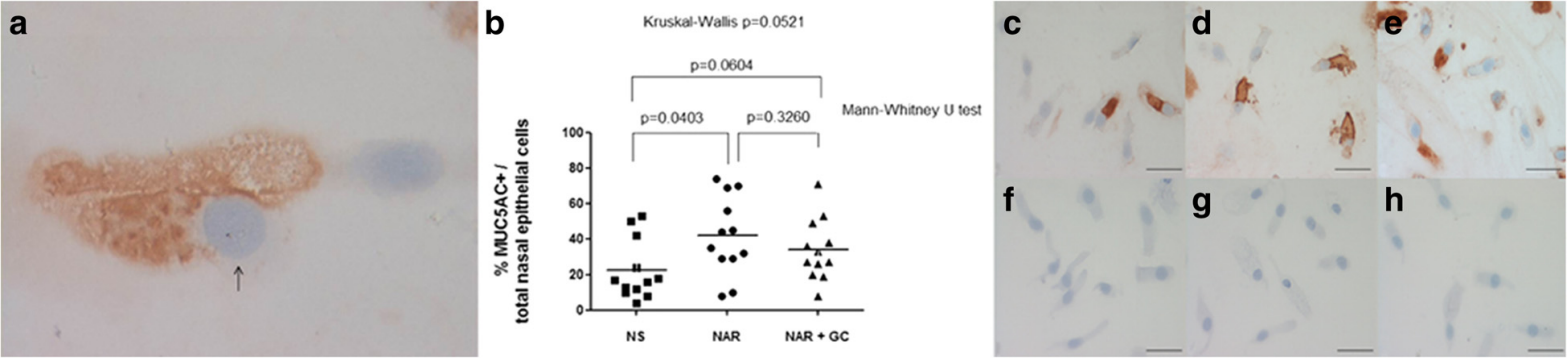
**a**) Photomicrograph showing a nasal epithelial cell immunostained for identification of MUC5AC+ cells. Nuclei are stained in *purple*/*blue* (*arrow*), cytoplasmic mucin MUC5AC is stained in *brown*. **b**) The percentage of MUC5AC+ nasal epithelial cells over the total number of nasal epithelial cells is significantly increased in the persistent untreated nonallergic rhinitis (NAR) patients compared both to control normal subjects (NS) and the persistent NAR patients under regular treatment with nasal glucocorticoids (NAR + GC), without any significant difference between control subjects and persistent NAR patients under regular treatment with nasal glucocorticoids. The immunocytochemical images in the right panel are representative of those from 12 control group subjects (**c**), 12 patients with persistent untreated NAR (**d**) and 12 patients with persistent NAR under regular treatment with nasal glucocorticoids (**e**). The images **f**, **g** and **h** are their representative negative controls (nonspecific mouse IgG). Original magnification: x400. Bar =50 μm

The immunoreactivity for MUC5AC in the nasal epithelial cells was localized to both goblet and non-goblet cells (Fig. 4a).

The percentage of MUC5AC+ nasal epithelial cells over the total number of nasal epithelial cells was significantly increased in untreated NAR patients compared to control normal subjects (41.8 ± 6.4 vs 22.3 ± 4.8 %, respectively; 95 % confidence interval 27.7─55.8 vs 11.6─32.9, respectively; Mann–Whitney U test *p* = 0.0403; one-way ANOVA Dunn’s multiple comparison test *p* > 0.05). There were no significant differences between control subjects and NAR patients on nasal FF (33.9 ± 5.0 %; 95 % confidence interval 23.0─44.8; Mann–Whitney U test *p* = 0.0604) nor between the 2 groups of persistent NAR subjects (Mann–Whitney U test *p* = 0.3260) (Kruskal-Wallis test *p* = 0.0521) (Fig. 4b-h).

### Nasal epithelial and inflammatory cells p65+ count

**Fig. 5:**
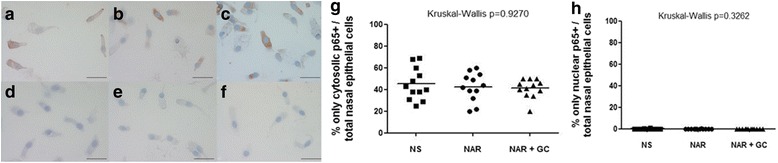
**a**) Photomicrographs showing nasal epithelial cells immunostained for identification of p65+ cells. Nuclei are stained in purple/blue, p65 is stained in *brown*. The immunocytochemical images in the *left* panel are representative of those from 12 control normal subjects (NS) (A), 12 patients with persistent untreated nonallergic rhinitis (NAR) patients (**b**) and 12 patients with persistent NAR patients under regular treatment with nasal glucocorticoids (NAR + GC) (**c**). The images **d**, **e** and **f** are the negative controls (nonspecific rabbit IgG). Original magnification: x400. Bar = 50 μm. The percentage of cytosolic only p65+ nasal epithelial cells (**g**) and nuclear p65+ nasal epithelial cells (**h**) over total nasal epithelial cells is not significantly different between persistent untreated NAR patients, NS and NAR + GC

p65+ nuclear and cytoplasmic staining was observed in nasal epithelial cells (Fig. 5a-f). There was no difference between any of the groups studied with respect to cytosolic and/or nuclear p65 staining (Fig. 5g and h). In detail, the percentage of cytosolic (42.5 ± 3.8 vs 45.2 ± 4.2 vs 41.2 ± 2.5 %, untreated persistent NAR patients vs control normal subjects vs NAR patients under regular FF treatment, respectively; 95 % confidence interval 34.2─50.8 vs 35.8─54.5 vs 35.7─46.7, respectively; Kruskal-Wallis test *p* = 0.9270) and nuclear (0.03 ± 0.03 vs 0.1 ± 0.09 vs 0.0 ± 0.0 %, untreated persistent NAR patients vs control normal subjects vs NAR patients under regular FF treatment, respectively; 95 % confidence interval −0.03─0.08 vs −0.07─0.3 vs 0.0─0.0, respectively; Kruskal-Wallis test *p* = 0.3262) p65+ nasal epithelial cells compared with the total number of nasal epithelial cells did not differ significantly between the 3 subject groups. In addition, no difference in these parameters were observed for the nasal inflammatory cells.

### Correlations between inflammatory cells and MUC5AC expression

There was no significant correlation between the number and/or type of inflammatory cells and MUC5AC expression in the nasal epithelial cells. In addition the outliers in the number of inflammatory cells and in the MUC5AC expression in the nasal epithelial cells were not the same patients.

## Discussion

We have shown here for the first time that patients with untreated persistent NAR, compared to control normal subjects, have an increased number of nasal inflammatory cells (both neutrophils and/or eosinophils) and nasal goblet cells and that the regular treatment with nasal FF for at least 20 days, is associated with a significant reduction in the number of these inflammatory cells as previously observed in controlled clinical trials performed in patients with chronic allergic rhinitis [10]. In addition we have also shown for the first time a significant increase in the number of MUC5AC+ nasal epithelial cells in patients with untreated persistent NAR, compared with control normal subjects, whereas their number is not significantly different between control subjects and persistent NAR patients under regular treatment with nasal FF.

The effect of the glucocorticoids on MUC5AC expression is still quite controversial. *In vitro* the effects of glucocorticoids on the expression of MUCAC appear cell-type dependent. For example, fluticasone propionate significantly reduced MUC5AC protein expression in the human lung mucoepidermoid carcinoma cell line (H292 cells) [11], whereas a treatment with dexamethasone did not change significantly the steady-state messenger ribonucleic acid (mRNA) levels of MUC5AC in cultured human nasal epithelial cells obtained from nasal polyps [12] and even enhanced both MUC5AC protein secretion and MUC5AC mRNA expression in cultured normal human bronchial epithelial cells [13–15]. In addition, in an animal model of rhinovirus infection fluticasone propionate increased MUC5AC protein level in the bronchoalveolar lavage [16].

Tang et al. reported a significant decrease in MUC5AC mRNA in human nasal epithelial cells and of MUC5AC expression in nasal mucosal biopsy specimens in patients with allergic rhinitis after treatment with fluticasone propionate (100 μg of nasal spray per nostril once daily for 4 weeks). In this report, patients with traumatic optic neuropathy ready for optic nerve decompression were enrolled as controls [17]. In contrast, nasal administration of glucocorticoids increased the level of mucin recovered by nasal lavage of non-atopic subjects. In this study, subjects were randomized to receive either 200 μg of fluticasone propionate once daily or 100 μg of beclomethasone dipropionate twice daily to one nostril and placebo to the other, chosen randomly [18].

These human data in nasal cells are also in keeping with other studies performed in the lower airways of asthmatic patients. For example Groneberg et al. reported no change in MUC5AC protein expression in the bronchial biopsies after treatment with inhaled budesonide of asthmatic patients [19]. Also Fahy et al. did not observe any significant effect on mucin-like glycoproteins in the sputum of moderate asthmatics by inhaled beclomethasone dipropionate [20]. The functional role of MUC5AC in the nasal inflammation is still unknown but animal data suggests that this secretory mucin is not involved in the immune defenses of the upper airways [21] despite its potential protective role during influenza infection of the lower airways [22].

We did not observe any significant difference in the nuclear (an index of activation) or cytosolic distribution and expression of p65, one of the main subunits of NF-kB, both in the nasal epithelial and inflammatory cells in all the three groups of subjects. The absence of nuclear translocation of p65 in all subjects suggests that there was no spontaneous NF-kB activation during the collection and processing of the samples. This suggests that pro-inflammatory gene transcription and MUC5AC transcription in these groups of subjects are regulated by other pro-inflammatory transcription factors or pathways. The definitive way to determine the role of NF-kB or of any other transcription factor in driving MUC5AC expression is to perform chromatin immunoprecipitation (ChIP) analysis in these samples. Samples were not available for this study and there are concerns that the sensitivity of the assay would not allow detection in the small number of cells available from each subject.

Our study has several limitations. Firstly, this was an open study and the effects of nasal FF must be confirmed in a controlled clinical trial using a placebo arm and a cross-over design. In addition this was a pilot study examining a small number of patients. We also acknowledge the limitation that the mean age of the group of patients with persistent NAR under regular treatment with nasal FF is significantly, albeit slightly, higher compared with the control group of the healthy subjects and this may have influenced the results.

In addition we did not have any objective confirmation of patient compliance to nasal FF treatment. However the presence of reduced nasal symptoms, better quality of life scores and a decreased number of inflammatory cells observed in nasal scrapings, in the group of patients treated with nasal FF compared to untreated patients, suggest that these patients were, at least partially, compliant with this treatment. In summary our findings suggest the great potential for the use of nasal scrapings in investigating the molecular pathogenesis of persistent non-allergic rhinitis.

## Conclusions

Patients with persistent untreated NAR, compared with normal control subjects and patients with persistent NAR under regular treatment with nasal fluticasone furoate by at least 20 days, have more nasal symptoms, worst quality of life and an increased number of nasal inflammatory cells (neutrophils, eosinophils), goblet cells and MUC5AC+ nasal epithelial cells. This nasal inflammation seems unrelated to NF-kB activation.

## Abbreviations

DAB, 3-3’-Diaminobenzidine; F, female; FF, fluticasone furoate; IgE, immunoglobulin E; M, male; MUC5AC, mucin 5 AC; NAR, nonallergic rhinitis; NF-kB, nuclear factor-kappa B; PBS, phosphate-buffered saline; PFA, paraformaldehyde; RAST, RadioAllergoSorbent test; RQLQ, Rhinoconjunctivitis Quality of Life Questionnaire; SEM, standard error of the mean
